# Reflection in the Context of the Epidemic: Does Death Anxiety Have a Positive Impact? The Role of Self-Improvement and Mental Resilience

**DOI:** 10.3389/fpsyg.2022.804635

**Published:** 2022-03-23

**Authors:** Yang Luo, Rui Guo, Chaohua Huang, Yan Xiong, Fei Zhou

**Affiliations:** School of Economics and Management, China University of Geosciences (Wuhan), Wuhan, China

**Keywords:** concept of justice and interests, death anxiety, environmental protection consumption, self-improvement, mental resilience

## Abstract

Public health emergencies can trigger individual death anxiety. Most previous studies focus on the negative effects of death anxiety *via* the Western materialistic view, neglecting both the positive aspects of death anxiety within the Chinese cultural background and the positive effects of death anxiety upon environmental consumption. By implementing the unique Chinese cultural background for the concepts of justice and interests, this study explores the positive influence of individual death anxiety on altruistic environmental consumption during the COVID-19 crisis by analyzing personal life reviews and other sources. The results show that (1) under the guidance of the correct concept of justice and benefit, individuals with high death anxiety during the epidemic period not only enhance their self-esteem through positive self-perception and social evaluation, but they are more inclined to benefit from other environmental consumption behaviors and attain a symbolic self-survival; and (2) during the epidemic period, mental resilience, as a transformation mechanism of external defense and the internal growth of death psychology, can directly affect altruistic environmental consumption by consumers without relying on external standards. In the context of the Chinese culture’s concept of justice and interests, this study enriches the knowledge of fear management theory and the positive impact of death anxiety on environmental consumption. The introduction of mental resilience as a boundary condition has important theoretical and practical significance within the study of consumer behavior in public health emergencies and post-epidemic economic recovery.

## Introduction

The spread of COVID-19 has greatly increased people’s cognitive and emotional experience of death and triggered a range of psychological behavioral characteristics. Terror Management Theory (TMT) suggests that death anxiety increases with the increase of threat sources, including COVID-19 (Courtney et al., 2020). This theory also points out that when people face the inevitability of death, they will activate specific psychological defense mechanisms, such as strengthening self-esteem and self-continuation to relieve the anxiety caused by the inevitability of death (Landau et al., 2004). At the level of self-perpetuation, some marketing scholars note that death anxiety can enhance individuals’ desire to buy products with environmental characteristics (Giannelloni, 1998), but from the perspective of Chinese cultural values, the application of death anxiety in the exploration of Chinese consumers’ behavior still needs to be strengthened. What is the impact of the correct values of justice and interests within Chinese culture on death anxiety during the epidemic? Can death anxiety drive green consumption? What is the effect of individual psychology on death anxiety? Can it be used as the transformation mechanism of external defense and for the internal growth of death psychology? These questions have received increasing attention from scholars in the research field of the psychology of death.

Although research on death anxiety has achieved fruitful results, there are still many shortcomings. First, death anxiety is common among people (Zhou et al., 2019a), including children, college students, and the elderly, which is affected by many factors such as gender, age, religious belief, region, self-esteem, personality characteristics, structural needs, self-control, parents’ health level, and attachment level (Fu and Guo, 2011; Guo, 2020)。Many other scholars have studied the death anxiety of critically ill and terminally ill patients. The diagnosis of terminally ill patients is a strong death reminder signal for patients, resulting in negative emotions such as death anxiety, fear, and depression, leading to serious psychological problems, self-esteem, self-efficacy, and good relationship with medical staff Better end-of-life preparation is a protective factor of death anxiety, which can alleviate patients’ death anxiety (Zhou et al., 2020; Miao et al., 2020) But current studies mainly focus on medical judgment and the treatment of death anxiety, as well as related social issues (e.g., Yang et al., 2013). These studies pay little attention to the impact of death anxiety on individual consumption behavior. Second, most previous studies have investigated on the negative impact of death anxiety with the understanding that it is a basic human anxiety hidden at the bottom of various psychological disturbances (Iverach and Menzies, 2014). This impact will cause individuals to experience negative emotions such as anxiety, fear, and sadness (Ferraro et al., 2005). However, according to other studies, death anxiety has the adaptive value of survival. In addition to the negative fear of death, psychological research concerning death also identifies positive death introspection (Duan, 2014). Finally, death anxiety is typically presented in the context of Western materialism. There are few studies regarding mainland subjects from a Chinese background. The “right approach to justice and interests” proposed by General Secretary Xi during his visit to Africa in 2013 has become an ethical value that has exerted great influence both at home and abroad. This “correct” concept of justice and interests includes the moral treatment of the relationship between public interests and personal interests and the concept of a harmonious coexistence between man and nature. Under the guidance of the correct concept of justice and interests, individuals with death anxiety value the significance that spiritual value has for people, treat public interests cautiously, and carry out environmental protection consumption. By implementing this Chinese cultural context, this study makes use of terror management theory, deeply explores the positive significance of death anxiety, discusses the role of self-improvement within death anxiety and upon altruistic environmental consumption, and elaborates the impact of mental resilience as an external defense of death psychology and an internal growth transformation mechanism on the above relationship.

## Theoretical Background and Hypotheses Development

### Death Anxiety and Altruistic Environmental Consumption

Death anxiety has consistently been a hot spot in foreign psychological research (Sliter et al., 2014). Mainstream studies believe that death anxiety is caused by the anticipation of one’s own death or by the negative emotions caused by meditation on one’s own death and the death of others. As the most systematic theory of death anxiety research, terror management theory holds that human beings’ self-subsisting instinct and their cognitive ability to perceive the inevitability of death make them anxious and always in a state of fear when facing death. From the relevant point of view of existing literature, death anxiety is all the negative emotional responses triggered by conscious (Neimeyer, 1998) or unconscious (Greenberg et al., 1990) knowledge related to self-extinction. This study was carried out during a major public health emergency. Individuals feel the severity of the epidemic through different channels and have empathy for the threat of death. Therefore, within this particular dimension, death anxiety is considered to include two parts: individual death anxiety and the death anxiety of others.

Since Greenberg et al. (1990) formally proposed terror management theory (TMT), death anxiety has become a new research field in consumer psychology. Within the psychology of death, people’s consumption psychology and behavior demonstrate some regular changes. The currently widely recognized defense mechanisms of death anxiety include the “world view defense” and the “enhancement of self-esteem” (e.g., Kastenmüller et al., 2013). Later research put forth a defense mechanism of death anxiety that is independent of worldview and self-esteem, called “self-perpetuating.” Erikson (1992) pays special attention to self-continuation in the time dimension and proposes the theory of “creation” to describe the behavior of individuals concerned about the welfare of their offspring. Creation here not only refers to material wealth but also includes intangible wealth, such as spirit and skills. Accordingly, environmental protection behavior can preserve complete natural assets for future generations and fulfill self-preservation. A few foreign scholars have verified the relationship between death anxiety and environmental protection behavior (e.g., Vess and Arndt, 2008), but there are few studies conducted within the Chinese cultural background. In this Chinese context, the concept of justice and interests refers to people’s cognition, understanding, and judgment of “justice” and “interest,” as well as the relationship between the two. “Justice” is altruistic and belongs to a noble moral principle, while “interest” is self-serving and generally refers to material interests (Li, 2016). This correct view of justice and interests therefore emphasizes that justice comes first; that is, it pays more attention to collective interests and has an inner spiritual consistency with the valued goal of harmonious coexistence.

Environmental consumption refers to people’s efforts to protect the ecological environment in the process of consumption and the pursuit of a minimum negative impact on the environment. Sustainable consumption behavior is primarily concerned with the nature of social responsibility (Lao, 2013). Some researchers believe that environmental consumption is influenced by altruistic values centered on the overall interests of society and self-centered egoistic values (Stern, 2000). Green consumption involves balancing one’s own costs/benefits against those of others. For example, green products cost more and are of poorer quality than ordinary products, but green products benefit everyone’s environment (Griskevicius et al., 2010). Therefore, it is believed that environmental protection requires personal cost, but it may not form personal benefit, but more for others and long-term benefit. In the long-term, from this perspective, environmental behavior can be understood as a prosocial behavior aligned with the traditional Chinese cultural concepts of justice and the interests of the group. Terror management theory holds that individuals with high death anxiety will exhibit prosocial behaviors and, furthermore, that individuals with high death anxiety will benefit from other environmental behaviors. However, the correct concept of justice and interests in the Chinese cultural context is shaped by an organic combination of collective interests and individual interests, an emphasis on the harmonious coexistence of humans and nature, a primary concern with long-term interests, and environmental protection consumption. As the unique national spirit and psychology of the Chinese nation, the correct concept of justice and interests can also inform the world defense of death anxiety to a certain extent.

Existentialist theory believes that death anxiety urges people to pursue the meaning of life more strongly. If we can correctly give shape to death anxiety, adhere to the correct concept of justice and interests, and seek the harmonious coexistence between man and nature, these factors will help guide people to create different life meanings driven by death anxiety. After experiencing major public health emergencies, individuals often have a strong death anxiety and tend to engage in altruistic environmental consumption to leave complete natural resources so that future generations may experience this symbolic inheritance for themselves.

Therefore, the following hypotheses are proposed in this study:

*H1*: Under the guidance of the correct concept of justice and profit, individuals with high death anxiety will engage in altruistic environmental consumption compared with individuals with low death anxiety.

### The Mediating Role of Self-Improvement Between Death Anxiety and Altruistic Environmental Consumption

All people in the world need to positively affirm themselves; this is a universal self-motivation shared by all human beings (Liu et al., 2011). Self-improvement is both an individual’s tendency to strive to maintain and improve self-esteem in self-comparison and the internal psychological driving force to seek positive identification and avoid negative evaluation (de Angelis et al., 2012). The drive for self-improvement is the need for self-esteem, and self-esteem is the basis of improving drive. According to the “buffer theory” of death anxiety, self-esteem is a mechanism to buffer death anxiety (Hart, 2014), and self-improvement is an important means to relieve anxiety. Therefore, individuals with death anxiety tend to have a higher need for self-improvement.

The psychology of death involves two types of responses: external defense and internal growth. Death anxiety is an external defense mechanism, which is people’s immediate reaction to the abstract concept of death (Wei et al., 2015). When the event gradually precipitates into a long-term reaction, people tend to reflect on the death of internal growth. In the process of such death reflection, consumers tend to pursue intrinsic value and exhibit prosocial motivations and behaviors (Wei et al., 2015). The self-improvement of Chinese people is usually characterized by the interpersonal and concealment. Chinese people tend not to publicly demonstrate the improvement of their sense of personal value. Instead, they improve themselves in relatively gentle ways, such as environmentally friendly consumption. The correct concept of justice and interests in Chinese culture emphasizes the significance of spiritual value more than human development, balancing spiritual pursuit with material desire. As a type of prosocial behavior, environmental consumption not only contains the principle of mutual benefit and improvement in the correct concept of justice and interests, but also embodies the spiritual pursuit of an ecological civilization.

This study argues that individuals with death anxiety relieve their own anxiety by improving their self-esteem, and self-improvement drives the need for self-esteem. To obtain an improved social evaluation and positive self-perception, consumers will tend to engage in altruistic environmental consumption. The defense of self-esteem is formed on the basis of social approval. Therefore, the measurement of self-improvement in this study includes both an individual’s self-perception and the social evaluation of individuals. Therefore, a second hypothesis is proposed in this study:

*H2*: Under the guidance of the correct concept of justice and profit, self-improvement plays a mediating role between death anxiety and altruistic environmental consumption.

### The Regulating Effect of Mental Resilience

External investigations of the defensive response suggest that the positive response to death anxiety is unstable because it is immediate and based on external criteria (Wei et al., 2015). This traditional concept of external defense is abstract, a lifeless review process from other perspectives. By contrast, this study includes mental resilience as a personal trait. People with high resilience do not rely on external criteria but prefer specific long-term responses, which gradually translate into internal growth.

Mental resilience refers to the process in which an individual can adapt well and develop actively after experiencing adversity (Masten, 2011). It is the ability of an individual to recover from negative experiences and flexibly adapt to changes in the external environment. Encountering adversity and coping successfully are the core elements of mental resilience (Lazarus, 1993). Block and Kremen (1996) elaborate how mentally resilient people are more adaptable and tolerant. They are willing to contribute to society and change themselves to become a better version of themselves.

Western psychology has been extensively researched in regard to mental resilience, and in Chinese classical culture, we see its origin. Zhouyi says: “Heaven is vigorous, the gentleman to unremitting self-improvement”; The Analects of Confucius write, “Beneficence is the first difficulty and then the second, which is called benevolence.” Terror management theory, however, developed within the context of Western culture. When discussing death anxiety in a Chinese context, it is necessary to consider the influence of Chinese culture. The ancients emphasized self-improvement and perseverance instead of clinging to the current difficulties and achieving a better self with a positive attitude. The ethical wisdom that buffers death anxiety is based on a belief in a world view, outlook on life, and values that transcend the symbolism of physical death and the eternity of life.

According to the protective model of mental resilience, although risk factors can have adverse effects on individual development, the interaction between mental resilience, risk factors, and negative emotions will reduce the possibility of negative consequences (Luthar et al., 2000). Kesebir (2014) challenges the hypothesis of TMT that “self-esteem alleviates fear of death,” believing that the root of alleviating death anxiety lies in facing death rather than denying and fearing it. A calm and open attitude toward death is an important condition for an individual’s internal growth, which is also the consistent view of PTG, NDErs, creationism, and other studies on the psychology of death.

Therefore, individuals with high mental resilience will treat death with a more positive attitude and have an increased ability to adapt to the external environment. During an epidemic, they recover quickly from negative experiences and are willing to contribute to society and change themselves for self-improvement. Emphasizing an improved living environment for future generations, as well as the realization of self-continuation and inheritance, they tend to engage in altruistic and environmentally friendly consumption. By contrast, for individuals with low mental resilience, it is difficult for them to adapt to the negative impact of the epidemic, and they are unable to have an open and calm attitude toward death. According to terror management theory, individuals with high death anxiety will have the motivation and behavior of self-protection withdrawal, and it is difficult for them to engage in altruistic environmental consumption. However, the morbidity of individuals with low death anxiety is not clear. Due to the effect of social norms and self-comparison, individuals will engage in altruistic environmental consumption based on the internal psychological driving force of seeking positive identification and avoiding negative evaluation. Therefore, a third hypothesis is proposed in this study:

*H3*: Under the guidance of the correct concept of justice and profit, mental resilience plays a moderating role between death anxiety and altruistic environmental consumption. When individuals possess high mental resilience, death anxiety will significantly enhance their pursuit of self-improvement and thus enhance their altruistic environmental consumption. When individuals have low mental resilience, death anxiety will negatively affect their pursuit of self-improvement, thus exerting an impact on altruistic environmental consumption.

**Figure 1:**
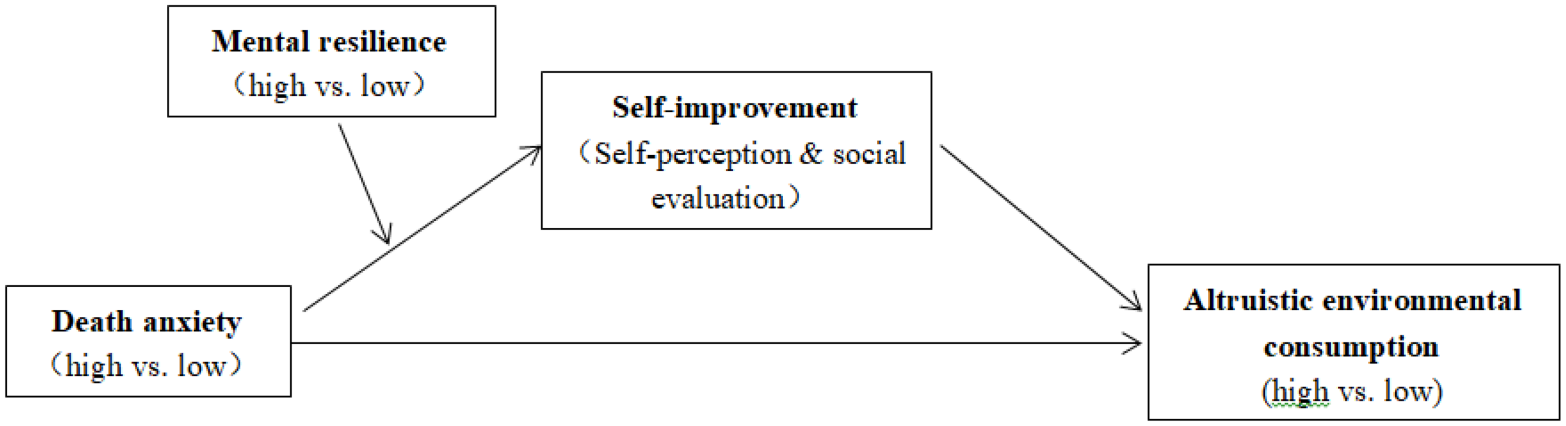
Research mode.

Figure 1 shows the model of this study.

## Methods and Results

Terror management theory advocates the experimental method to study the psychology of death and to arouse the awareness of death by examining the individual’s mortality salience (MS). In this study, death anxiety is a total of negative emotional responses triggered by conscious or unconscious knowledge related to self-extinction. As a result, this study of experiment method and the traditional MS differs slightly, although we also use two ways to answer the question and the real setting. This time, however, the setting was no longer artificially constructed, but rather in the period of COVID-19. Major outbreaks among individuals in a relatively dangerous situation, the individual’s practical experience, the government’s response, and the spread of network information, etc., were all death-related messages to consumers. These consumers experienced real security threats, instead of the traditional process of death. According to one survey, Chinese people paid high attention to the epidemic, with 58.93% saying they were very concerned about the epidemic, and 35.46% saying they were relatively concerned about the epidemic. More than 70% of the residents said they will continue to follow the latest news of COVID-19 through different channels.

This study was carried out in the context of the Chinese cultural concept of correct justice and interests. Physical surveys could not be conducted during the epidemic period, but some studies have shown that there is no significant difference between the results of online surveys and traditional paper questionnaires (Wen et al., 2020). Therefore, in this experiment, conducted between February and May of 2020, through the Questionnaire Star platform in China, village residents in enterprises such as statistical temperature groups distributed electronic questionnaires. The group coverage included relatively scattered distribution areas, occupations, economic structures, and education levels that were diversiform and accurately reflected public health emergencies during the outbreak of the majority group. The participants in this research received a certain share of a prize at the end of the research to increase their enthusiasm. The questionnaire opened by asking, “Do you feel the threat of death in this epidemic, and therefore feel anxious?” If the answer was “Yes,” then the questionnaire was valid and statistically analyzed. If the answer was “no,” then it was removed as an invalid questionnaire.

Study 1 initially verified Hypothesis 1 and proved the positive effect of death anxiety on environmental consumption. Study 2 further verified Hypothesis 1 and verified Hypothesis 2 by demonstrating the mediating role of self-improvement between death anxiety and altruistic environmental consumption. In Study 3, Hypothesis 3 was verified by a two-factor intergroup experiment of 2 (death anxiety: high vs. low) × 2 (mental resilience: high vs. low); namely, mental resilience played a moderating role in the relationship between death anxiety and altruistic environmental consumption.

### Study 1

Study 1 verified Hypothesis 1 through a unifactor intergroup experiment, which confirmed our hypothesis; namely, under the guidance of the correct concept of justice and benefit, individuals with high death anxiety showed more active environmental consumption behavior.

#### Method

##### Participants and Design

The researchers recruited 172 participants through the questionnaire star platform. One participant had the same answers, three had less than 5 min to answer questions, and two guessed the true purpose of the survey. These six questionnaires were deleted as unqualified questionnaires, and finally, 166 valid questionnaires were obtained. Among them, 93 are male and 73 are female, with an average age of 27.9 years. During the study, we allowed the participants to browse secretary Xi’s explanation of the correct concept of justice and interests, let them review nearly a week of China’s accumulative total number of confirmed cases, arousing their anxiety about death, and then let the participants complete the death anxiety questionnaire. Based on the average (M_death anxiety_ = 2.96), the participants were divided into high death anxiety and low death anxiety groups. Finally, the subjects were asked to complete the measurement of environmental consumption.

##### Measurement of Death Anxiety

Earlier research mostly argued that death anxiety is a kind of fear of death, because this often used a single dimension to measure it (Zhou et al., 2019b), but with the development of the theory and practice, researchers gradually realized that people’s fear of death is complex. Death anxiety being defined according to the different aspects of death is more scientific and appropriate. This study is a localized study of Death anxiety for Chinese people in the context of Chinese culture, using the Chinese Version of Templer-Death Anxiety Scale, Ct-das was measured in combination with the Short Health Anxiety Inventory (SHAI) and the current status of COVID-19. The CT-DAS scale originates from the Templer-Death Anxiety Scales (T-DAS) and was published by Professor Templer (1970). It is a self-rated multidimensional scale. Yang et al. (2013) examined the applicability of DAS in China. In this study, CT-DAS scoring was set to Likert 5 according to the standard of the subsequent T-DAS version (Templer et al., 2006). Referring to the treatment of death anxiety variables by Chinese scholars, death anxiety was divided into high death anxiety group and low death anxiety group according to the mean value (Yang et al., 2013; Zhou et al., 2019a). In the implementation process, the description of disease in the original death anxiety scale was replaced by the description of COVID-19, and the evaluation of personal and mental health was added. A total of 12 items were included, of which seven items were scored forward and five items were scored backward.

##### Measurement of Environmental Consumption

This study referred to the studies of Kronrod et al. (2012) and (Xiong, 2015) on green consumption and asked consumers about their attitudes and intentions to engage in environmentally friendly consumption. The measurement includes two items: ① Considering environmental factors, I would like to choose greener products after the epidemic is over; ② I would like to recommend this environmentally friendly product to my friends. This test item used a 5-level scale, with 1 representing “strongly disagree” and 5 representing “strongly agree.” These two test items were used to measure consumers’ willingness to consume environmental protection to preliminarily verify Hypothesis 1.

#### Results and Discussion

ANOVA for 2 (death anxiety: high vs. low) × 2 (environmental consumption: high vs. low) showed a significant two-way interaction [*F*(1,165) = 20.74, *p* < 0.001]. Consistent with Hypothesis 1, in the cultural context of correct values of justice and interests, people with high death anxiety were more inclined to engage in environmental consumption than those with low death anxiety (M_high death anxiety_ = 3.96, SD = 0.77 vs. M_low death anxiety_ = 3.42, SD = 0.73; *F*(1,165) = 20.74, *p* < 0.001, *d* = 0.72).

In the event of a major public health emergency, consumers perceived the death threat, took environmental consumption as a self-sustaining inheritance mechanism, and tended to engage in environmental consumption. Hypothesis 1 had been preliminarily verified.

### Study 2

Study 2 aimed to further verify the influence of death anxiety on altruistic environmental consumption under the cultural background of the correct concept of justice and interests (H1). At the same time, the role of self-improvement in the above mechanisms was also verified (H2).

#### Method

##### Participants and Design

The researchers recruited 193 participants through the questionnaire star platform, three of whom gave the same answers, three of whom answered for less than 5 min, and five of whom guessed the true purpose of the survey. These 11 questionnaires were deleted as unqualified questionnaires, and 182 valid questionnaires were finally obtained. There were 87 males and 95 females with an average age of 30.2 years. During the experiment, we allowed the participants to browse secretary Xi’s explanation of the correct concept of justice and interests, let them review nearly a week of China’s accumulative total number of confirmed cases, arousing their anxiety about death, and then let the participants complete the death anxiety questionnaire, according to the average (M_death anxiety_ = 2.90). The participants were then divided into high death anxiety and low death anxiety group. Finally, the participants were asked to complete the measurement of self-improvement and altruistic environmental consumption.

##### Measurement of Variables

Death anxiety followed the scale of Study 1, in which the relationship between death anxiety and environmental consumption was measured. Here, the relationship between death anxiety and altruistic environmental consumption was further explored.

Environmental protection consumption is influenced by the “concept of justice and benefit” in traditional Chinese culture, which has the two tendencies of self-interest and altruism. This study only explores the relationship between death anxiety and altruistic environmental protection consumption. In reference to the studies of Kronrod et al. (2012) and (Xiong, 2015) on green consumption, the measurement of altruistic environmental consumption contains two items: ① I purchase this product based on the consideration of environmental protection compared with the consideration of personal health; ② Compared with the consideration of product value, I purchase this product based on the consideration of maintaining ecological balance. This test item used a five-level scale, with 1 representing “strongly disagree” and 5 representing “strongly agree.”

In this study, a self-improvement scale (four items) developed by scholars such as Engel et al. (1995) was adopted, self-improvement measurement was made with reference to Sun and Guo (2016), and appropriate adjustments were made based on current research. ① In my opinion, a series of environmentally friendly consumption behaviors can enhance my influence in my circle of friends; ② I believe that by carrying out a series of environmentally friendly consumption behaviors, I can gain recognition and appreciation from others; ③ I think that a series of environmentally friendly consumption behaviors can show that I am a person who loves life, the environment and is responsible; and ④ In my opinion, carrying out a series of environmentally friendly consumption behaviors can improve my personal image. A five-point scale was used, with 1 representing “strongly disagree” and 5 representing “strongly agree.”

#### Results and Discussion

##### Self-Improvement

One-way ANOVA results showed that the self-improvement of the high death anxiety group was significantly higher than that of the low death anxiety group (M_high death anxiety_ = 3.55, SD = 0.80 vs. M_low death anxiety_ = 2.87, SD = 0.70; *F*(1,181) = 36.81, *p* < 0.001, *d* = 0.90).

##### Altruistic and Environmentally Friendly Consumption

One-way ANOVA results showed that the altruistic environmental consumption of the high death anxiety group was significantly higher than that of the low death anxiety group [M_high death anxiety_ = 3.93, SD = 0.87 vs. M_low death anxiety_ = 3.44, SD = 0.78; *F*(1,181) = 15.96, *p* < 0.001, *d* = 0.60].

##### Mediation Analysis

According to the suggestion of Hayes (2013), the bootstrap method was used in this paper to test the mediating role of self-improvement between death anxiety and altruistic environmental consumption. The results showed that the mediating effect of self-improvement was significant (95% confidence interval *β* = 0.27; CI = 0.151 to 0.405). In addition, after controlling for the mediating variable (self-improvement), the effect of death anxiety on altruistic environmental consumption became insignificant (95% confidence interval *β* = 0.22; CI = −0.029 to 0.469). Therefore, H2 is verified as: death anxiety affects altruistic environmental consumption through self-improvement as a mediating factor.

Study 2 shows that, in the context of the correct concept of justice and interests in China, compared to individuals with low death anxiety, individuals with high death anxiety are more likely to favor other environmental consumption behaviors, and self-improvement plays a mediating role in the above mechanisms, verifying H1 and H2.

### Study 3

The purpose of Study 3 was to examine the effect of mental resilience on the relationship between death anxiety and altruistic environmental consumption through an intergroup design of 2 (death anxiety: high death anxiety and low death anxiety) × 2 (mental resilience: high mental resilience and low mental resilience; H3).

#### Method

Figures, tables, and images will be published under a Creative Commons CC-BY license and permission must be obtained for use of copyrighted material from other sources (including re-published/adapted/modified/partial figures and images from the internet). It is the responsibility of the authors to acquire the licenses, to follow any citation instructions requested by third-party rights holders, and cover any supplementary charges.

##### Participants and Design

The researchers recruited 305 participants through the questionnaire star platform, four of whom gave the same answers, six of whom answered for less than 5 min, and one of whom guessed the true purpose of the survey. These 11 questionnaires were deleted as unqualified questionnaires, and 294 valid questionnaires were finally obtained. Among them, 133 are male and 161 are female, with an average age of 29.7. Experimenters allowed the participants to browse secretary Xi’s explanation of the correct concept of justice and interests, let them review nearly a week China’s accumulative total number of confirmed cases, arousing their anxiety for death, and then let the participants complete the death anxiety questionnaire. According to the average (M_death anxiety_ = 3.08), they were divided into a high death anxiety and low death anxiety group. Finally, the participants were asked to complete measures of self-improvement, mental resilience, and altruistic environmental consumption.

##### Measurement of Variables

Death anxiety, self-promotion, and altruistic environmental consumption followed the scales in Study 2.

##### Measurement of Mental Resilience

The Connor-Davidson Resilience Scale (CD-RISC; Laura, 2007) was adopted. The scale includes 10 items, and five grades are used, all of which are positive test items, with 1 representing “never” and 5 representing “always.”

#### Results and Discussion

##### Main Effect Analysis

Statistical results showed that the main effect of death anxiety on altruistic environmental consumption was significantly different [*F*(1,293) =2.88, *p* < 0.001], and H1 was verified again. The marginal main effect of individual mental resilience on altruistic environmental consumption was also significant [*F*(1,293) = 2.07, *p* < 0.001].

##### Interaction Analysis

Altruistic environmental consumption as the dependent variable, to 2 (death anxiety: high vs. low) × 2 (resilience: high vs. low) analysis of variance, the results showed that the death anxiety and mental flexibility altruism are significantly influenced by the interaction of environmental consumption [*F*(1,293) = 2.73, *p* < 0.001].

##### Simple Effect Analysis

**Figure 2:**
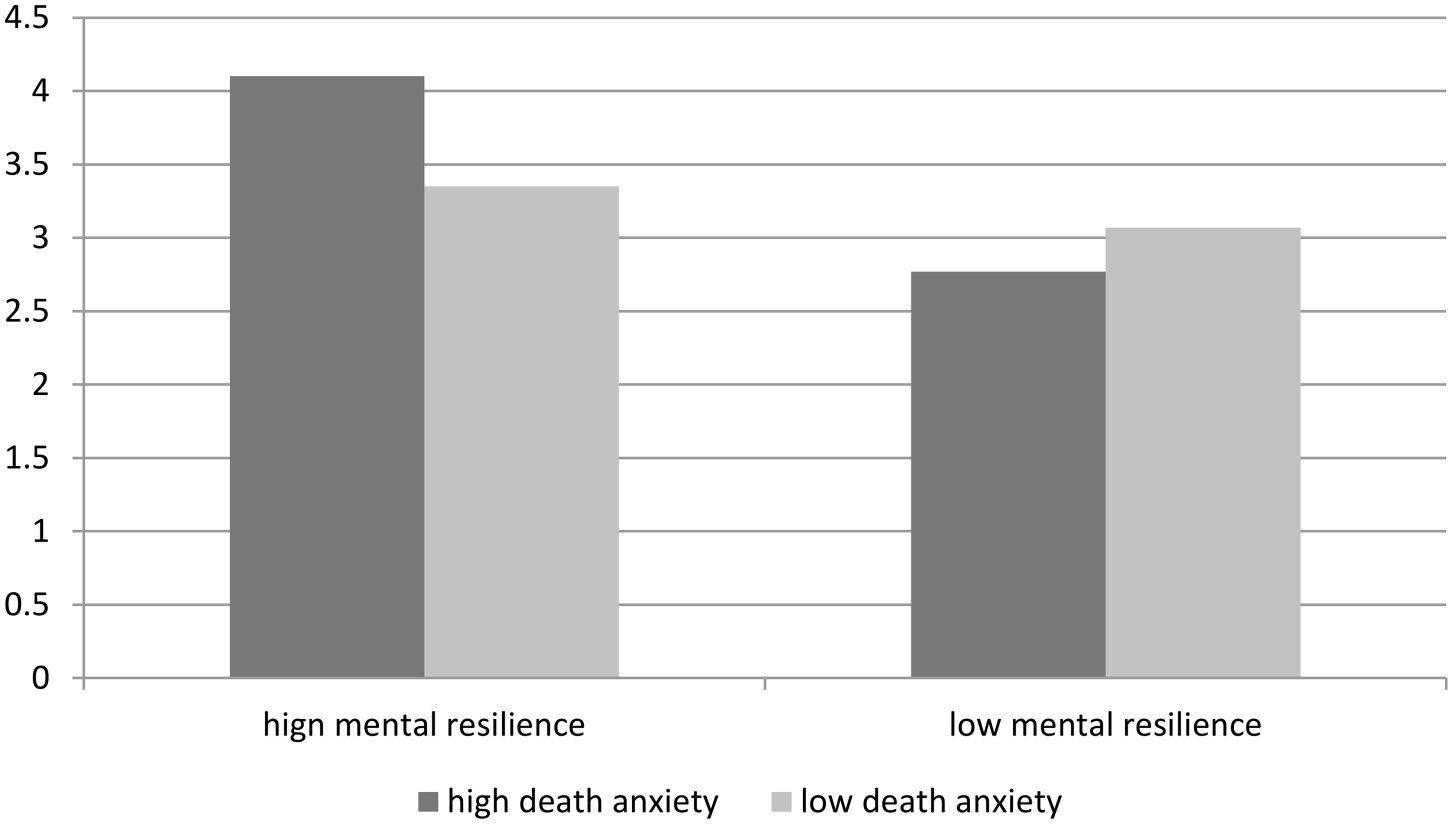
The influence of the interaction between death anxiety and mental resilience on altruistic environmental consumption.

Based on the proof of a significant main effect, marginal main effect, and interaction effect, the subjects were divided into a high resilience group and a low resilience group according to the average score of mental resilience (M_mental resilience_ = 3.44). Participants were assigned to 2 (death anxiety: high vs. low) × 2 (resilience: high vs. low) experiments for simple effect analysis. The sample size of each group was N_high death anxiety, high mental resilience_ = 72; N_high death anxiety, low mental resilience_ = 73; N_low death anxiety, high mental resilience_ = 74; and N_low death anxiety, low mental resilience_ = 75. Further simple effect analysis results are shown in Figure 2. In the case of high mental resilience, death anxiety significantly affected altruistic environmental consumption (M_high death anxiety_ = 4.10, SD = 0.73 vs. M_low death anxiety_ = 3.35, SD = 0.72, *d* = 1.03; *F*(1,145) = 39.27, *p* < 0.001); in the case of low mental resilience, death anxiety significantly affected altruistic environmental consumption (M_high death anxiety_ = 2.77, SD = 0.61 vs. M_low death anxiety_ = 3.07, SD = 0.64, D = 0.48; *F*(1,146) = 8.09, *p* < 0.05).

##### Mediating Effect Analysis

**Figure 3:**
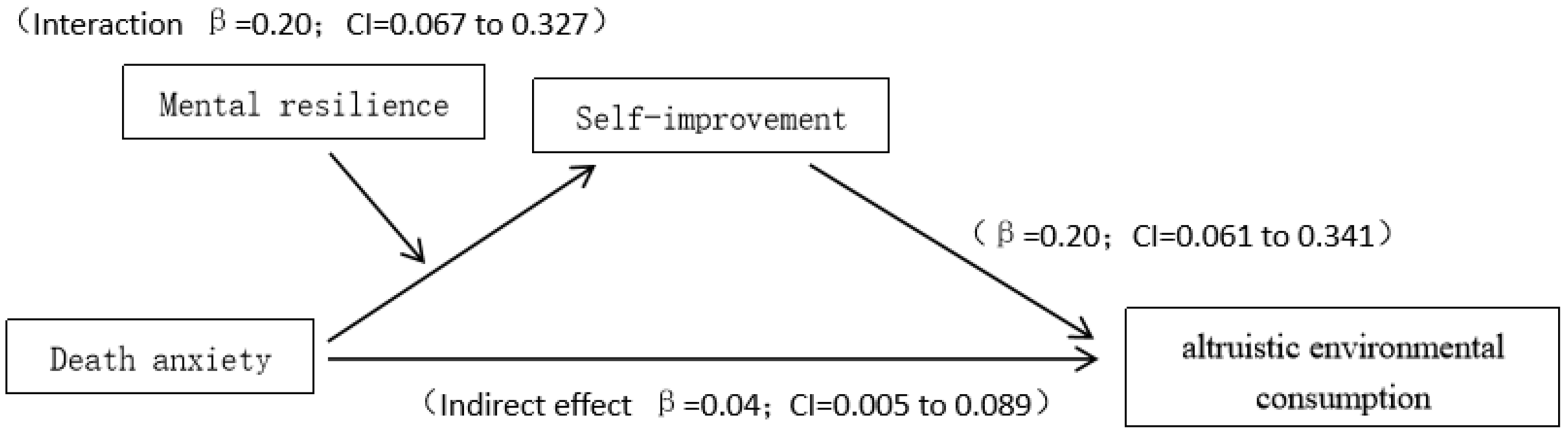
Mediating effect analysis.

In this study, mental resilience (the moderating variable) moderated the relationship between individual death anxiety and altruistic environmental consumption by affecting self-improvement (the mediating variable). Therefore, this study adopted bootstrapping (Process Model 8, Hayes, 2013) to analyze the mediating effect of mental resilience. The results showed that the interaction between individual death anxiety and mental resilience could significantly affect individual self-improvement (95% confidence interval *β* = 0.20; CI = 0.07 to 0.33), and self-improvement had a significant effect on individuals’ altruistic environmental consumption (95% confidence interval *β* = 0.20; CI = 0.07 to 0.34). The results verified that individual mental resilience could effectively regulate the relationship between death anxiety and altruistic environmental consumption through self-promotion (95% confidence interval *β* = 0.04; CI = 0.00 to 0.09). See Figure 3 for details.

The experiment demonstrates that individual mental resilience can effectively regulate the impact of individual death anxiety on altruistic environmental consumption, which verifies Hypothesis 3. When individuals have high mental resilience, death anxiety will significantly affect their altruistic environmental consumption. In other words, individuals with high death anxiety will tend to engage in altruistic environmental consumption due to their needs for inheritance, self-continuation, and self-improvement. When individuals have low mental resilience, death anxiety has an inhibitory effect on altruistic environmental consumption; that is, because of negative emotions, individuals with high death anxiety have more difficulty engaging in altruistic environmental consumption, while individuals with low death anxiety are more inclined to engage in altruistic environmental consumption due to their calmness of mind, the effect of social norms, and self-improvement.

### Discussion

Through different data analysis methods, the experiment verified the direct impact of high death anxiety on altruistic environmental consumption, and self-improvement played a mediating role. The experimental results again verified the inheritance motivation of death psychology and confirmed the correctness of creationism theory in the context of Chinese culture. When individuals have high death anxiety, under the guidance of the correct concept of justice and profit, they pay attention to the long-term interests of society and hope to realize a symbolic inheritance of the self by protecting the environment to carry out altruistic environmental consumption. To obtain better social evaluation and positive self-perception, consumers tend to engage in altruistic environmental consumption. That is, self-improvement plays a mediating role in the above mechanism.

More importantly, the experiment also verified the moderating effect of mental resilience on the above mediating effect; that is, for individuals with high mental resilience, death anxiety has a positive effect on altruistic environmental consumption. However, for individuals with low mental resilience, death anxiety has a negative effect on altruistic environmental consumption. This also explains why different groups have different consumption behaviors under similar social conditions and cultural backgrounds. This further explains how death anxiety affects altruistic environmental consumption through self-improvement by discussing the regulating effect of mental resilience. Therefore, the introduction of this regulating variable is in line with the empirical results expected by the theory and provides strong evidence for the applicability and accuracy of the experimental results.

## General Discussion

### Conclusion

Under the cultural background of the concept of justice and interests in China, this study focuses on the influencing mechanism of consumers’ death anxiety in the event of public health emergencies. Based on the terror of management theory, this study confirms the mechanism and boundary conditions between death anxiety and altruistic environmental consumption. The theory of terror management holds that when individuals have thoughts of self-destruction, they take steps to preserve themselves as much as possible. At present, there are four widely recognized symbolic mechanisms of self-perpetuation. The first is to realize self-affirmation through the defense of one’s cultural world outlook, thus realizing spiritual immortality. Second, we should regulate our behavior through the world outlook and values of the culture we belong to in order to enhance our self-esteem. Third, inheritance; that is, individuals pay attention to the welfare of their offspring through different forms. Fourth, by establishing and maintaining close relationships with others, the mechanism of seeking togetherness, intimacy, attachment, and connection with others is used to relieve anxiety (Mikulincer et al., 2003).

Based on the above questions and reflections, this paper explores the relationship between individual death anxiety, self-improvement and altruistic environmental consumption, as well as the moderating effect of mental resilience under public health emergencies. The study supported the assumptions and reached two important conclusions.

First, the perception of death anxiety will indeed lead to the pursuit of self-improvement, and then enhance the altruistic environmental consumption of individuals. Self-improvement is an important intermediary between death anxiety and altruistic environmental consumption. The need for self-improvement positively influences altruistic environmental consumption, which is a key indicator of why death anxious individuals enhance altruistic environmental consumption. Humans have a self-perpetuating instinct to take various defensive actions to relieve death anxiety. Whether it is the defense of their cultural world view, enhancement of self-esteem, inheritance or intimate relationship, it all shows that people are not simple individuals. They need to find their own value in social relations, actively improve their self-perception and others’ positive evaluation of them and realize self-continuation through symbolic mechanisms. The concept of justice and interests in Chinese culture focuses on long-term interests and collective interests, and emphasizes the harmonious coexistence between humans and nature. Altruistic environmental consumption is of great significance to sustainable development and can preserve relatively complete natural heritage and cultural value for future generations. Combined with this new epidemic, it is caused to some extent by the disharmony between humans and nature. In this case, environmental protection consumption is particularly important.

Second, on the path of the increase of altruistic environmental consumption caused by individual death anxiety, mental resilience is a key contributing factor for individuals to feel positive emotions and engage in altruistic environmental consumption. This factor is also an important influencing factor for the transition between external defense and internal growth in the psychology of death. Research on the psychology of death can be roughly divided into two categories: one focuses on negative death fear behavior; the other focuses on positive death acceptance, psychological growth, and prosocial response (Wei et al., 2015). The concept of death anxiety also has both positive and negative components, indicating that the outcome of death anxiety is not constant. In essence, studies on near-death, trauma and near-death experience in the psychology of death argue that death is terrible, and people’s psychology of facing death is also regarded as “whether or not” and negative. However, the psychology of death is a complex emotion with individual differences. When individuals blindly indulge in the negative emotions brought about by the inevitability of death (Duan, 2014), it will be difficult for them to experience psychological growth. By contrast, an individual with a better ability to adapt and recover from a bad situation tends to view the inevitability of death in a positive way, causing a kind of consciousness that can be called death introspection. In the context of death introspection, people will turn from the pursuit of external value to the pursuit of intrinsic and transcendental value. Individuals are also inclined to make contributions to society and to better keep in touch with others. Once their death anxiety is aroused, individuals with high mental resilience are more likely to feel positive emotions, make efforts to improve self-perception and social evaluation, and are more likely to form altruistic environmental consumption under this psychological mechanism in order to realize the enhancement of self-esteem and symbolic self-extension.

### Theoretical Contributions

First, this study expands the localization of death anxiety. Death anxiety and consumer behavior research have been faced with the challenge of cultural differences. Culture is a core concept in terror management theory, but the individual’s perception of death and feelings are influenced by different cultural backgrounds. Much of the existing research concerning death anxiety is built on the basis of the Western material culture, in the context of foreign empirical research conducted overseas, for overseas participants. Some scholars have conducted cross-cultural research (e.g., Kashima et al., 2004), but these studies did not go far enough. Therefore, it is necessary to study the effect of death anxiety on altruistic environmental consumption in Chinese subjects from the perspective of a Chinese cultural value—the concept of correct justice and benefit. The concept of correct justice and interests focuses on the correct relationship between “justice” and “interest” and adheres to sustainable development. In this cultural context, individuals have a deeper understanding of “justice” and “interest” and tend to adhere to altruistic environmental protection behaviors. This study is helpful to understand the death psychology of Chinese consumers, to promote the localization of the adaptation mechanism of death anxiety, and to explore the cultural differences of the impact of the death anxiety mechanism on consumer behavior.

Second, this study expands the research on consumer psychology in the positive aspect of death anxiety and explores the mechanism of external defense and internal growth of death psychology. Duan (2014), after integrating the studies of many researchers, believed that death anxiety was a negative emotional reaction triggered by knowledge related to self-extinction. However, studies on the psychology of death also include PTG, NDERS, creationism theory, and death introspection, all of which focus on the positive effects of the psychology of death. Building on the theories of fear management and creation, this study focuses on the positive effects of self-improvement on death anxiety from the perspective of personal life reviews and other people’s perspectives (i.e., social evaluation in self-improvement) and explores the long-term consumer behavior and prosocial motivation caused by death anxiety. This paper preliminarily explores the mechanism of external defense and internal growth of death psychology, identifies a psychological defense mechanism other than world view, self-esteem, and intimate relationships, and enriches the research concerning the positive responses of death anxiety. At the same time, this study also sheds light on the impact of individual mental resilience on individuals with death anxiety. The research shows that mental resilience regulates the relationship between death anxiety and altruistic environmental consumption and can improve the existing defense mechanism system of fear management theory.

The relationship between positive emotions and mental resilience has always been a hotspot of resilience research (Lyu et al., 2017). Most domestic research objects on mental resilience are high-risk groups, and the lack of resilience studies focusing on normal people and daily life situations results in limited research results and lack of universality. In this study, such studies were conducted on ordinary residents during the epidemic, which represented the state of the majority of Chinese consumers. Most of the subjects had anxiety during the epidemic, but they were not infected with COVID-19, which is helpful to understand the psychological state of the Chinese population in public health emergencies.

Third, this study enriches the research on death anxiety and altruistic environmental consumption behavior. Existing studies on death anxiety are mainly applied to medicine and psychology, less to consumer behavior, and even less to empirical studies on death anxiety and environmental consumption. Relevant studies have shown that some consumption behaviors, as symbols, can subconsciously help consumers relieve death anxiety; for example, a stronger demand for luxuries and an increased tendency to buy domestic products. However, there are few studies on death anxiety and environmental consumption. In previous studies, the impact factors of environmental consumption were divided into two categories: the social macro level and the individual level. The individual level includes gender, personal income, postmaterialistic values, knowledge, and education level (Dunlap and Yolk, 2008). Few researchers have considered the impact of emotion on environmental consumption. This research, according to terror management theory and the creation concept, suggests that consumers experiencing death anxiety tend to engage in self-esteem and inherit the defense mechanism to alleviate anxiety. Altruism environmental consumption helps individuals to obtain a more positive self-awareness and social evaluation; therefore, starting an experience of death anxiety due to altruism is a higher environmental consumption motive. This study enriches the research concerning death anxiety and environmental consumption behavior and expands knowledge regarding the initiation mechanism of green consumption.

Fourth, this study improved the initiation mode of death anxiety. The existing TMT uses MS to initiate mental death, which is mainly comprised of answering questions, using real scenes, and using subthreshold stimulation. All of these are laboratory experiments in which the subjects are asked to view pictures and videos and listen to audio related to death or to discuss death-related topics to arouse the subjects’ death anxiety. By contrast, this study was conducted when COVID-19 was prevalent. Due to the severity of the epidemic and the development of the Internet, the public has paid more attention to COVID-19. Most of the subjects were anxious about their health, which was slightly different from the previous MS start-up method.

### Practical Implications

This study provides a valuable reference for alleviating consumers’ death anxiety and promoting environmental consumption in the context of public health emergencies.

First, anxiety about death can have a significant impact on physical and mental health and must be properly managed by Governments and other agencies. Death anxiety is at its highest during the height of the epidemic and measures must be taken to mitigate the negative impact on health. In the face of such public health emergencies, attention should be paid to individual psychological changes, the dissemination of the correct concept of justice and interests, education, public opinion guidance and other ways to improve people’s ability to cope with and resist pressure. In the case of public health emergencies, we should pay attention to disease management and postdisaster reconstruction and psychological counseling of the masses, as well as spread correct values, form world view defense, and avoid long-term psychological problems. At the same time, quality education, media publicity, and other methods should be adopted to improve the individual’s mental resilience and improve the ability to cope with emergencies.

Second, based on the outbreak of COVID-19, management departments, media, and environmental protection departments should vigorously publicize environmental protection knowledge, carry out environmental protection education, and promote green production and consumption. Ecological progress is an important part of the cause of socialism in Chinese characteristics. This progress rests on the two centenary goals and the realization of the Chinese dream of national rejuvenation. Individuals tend to use altruistic environmental consumption as a relief mechanism of death anxiety, which is not only beneficial to the ecological environment but can also alleviate death anxiety. Vigorous publicity by relevant departments and media will stabilize this awareness and provide a beneficial way for the masses to relieve death anxiety, which is of great significance to the improvement of the ecological environment and the psychological guidance of the masses.

Third, in such public health emergencies, enterprises can provide prosocial products and services that represent environmental protection attributes. With the passage of time, individuals’ initial fear of death will transform into their internal growth. To pursue self-value improvement and social evaluation, individuals tend to adopt prosocial behaviors, while altruistic environmental consumption can satisfy consumers’ prosocial tendencies and instantiate symbolic inheritance. This is of great significance for enhancing the purchase intention of environmental protection products, promoting the construction of an ecological civilization, and promoting economic recovery after major epidemics.

### Future Research Directions

First, this study mainly focuses on the affected groups during the new crown period. The population tends to be younger with a relatively high educational level. The experimental setting and the selection of subjects may have had a certain influence on the final results. Follow-up research can expand the sample size and sample scope and to conduct experimental research on people of different ages, education levels, income levels, and even occupations to expand the universality and generalization of this research.

Second, this study focuses on the mechanisms of death anxiety and does not discuss the components of death anxiety in the context of public health emergencies. In future, studies might explore, in public health emergencies, which specific aspect influences consumer’s death anxiety compared to the existing death anxiety dimension; that is, what are the similarities and differences? Perhaps, these can be even further removed from existing death anxiety studies to explore the mechanism of individual death anxiety within cultural conditions to expand the overall knowledge of death anxiety and fear management theory.

## Data Availability Statement

The raw data supporting the conclusions of this article will be made available by the authors, without undue reservation.

## Ethics Statement

The studies involving human participants were reviewed and approved by China University of Geosciences, Wuhan. The patients/participants provided their written informed consent to participate in this study.

## Author Contributions

YL identified the problem area, built a hypothesis and worked on the significance of study. RG and CH contributed to the results and discussion chapter. All authors contributed to the article and approved the submitted version.

## Funding

This research was funded by The key project of National Natural Science Foundation of China (Grant No. 71532011), National Natural Science Foundation of China “Brand internationalization strategy and influence mechanism of Chinese national brands from the perspective of brand confidence” (Grant No. 71772168), and Key project of Jewelry Inheritance and Innovation Development Research Center (Grant No. CJHIXM-01-202001).

## Conflict of Interest

The authors declare that the research was conducted in the absence of any commercial or financial relationships that could be construed as a potential conflict of interest.

## Publisher’s Note

All claims expressed in this article are solely those of the authors and do not necessarily represent those of their affiliated organizations, or those of the publisher, the editors and the reviewers. Any product that may be evaluated in this article, or claim that may be made by its manufacturer, is not guaranteed or endorsed by the publisher.
